# scSNV-seq: high-throughput phenotyping of single nucleotide variants by coupled single-cell genotyping and transcriptomics

**DOI:** 10.1186/s13059-024-03169-y

**Published:** 2024-01-15

**Authors:** Sarah E. Cooper, Matthew A. Coelho, Magdalena E. Strauss, Aleksander M. Gontarczyk, Qianxin Wu, Mathew J. Garnett, John C. Marioni, Andrew R. Bassett

**Affiliations:** 1 Cellular and Gene Editing Research, Wellcome Sanger Institute, Hinxton, Cambridge, CB10 1SA UK; 2 Translational Cancer Genomics, Wellcome Sanger Institute, Hinxton, Cambridge, CB10 1SA UK; 3 Open Targets, Wellcome Genome Campus, Hinxton, Cambridge, CB10 1SD UK; 4 EMBL-European Bioinformatics Institute, Wellcome Genome Campus, Hinxton, Cambridge, CB10 1SD UK; 5 Cellular Genetics, Wellcome Genome Campus, Hinxton, Cambridge, CB10 1SD UK; 6 Cancer Research UK Cambridge Institute, University of Cambridge, Robinson Way, Cambridge, CB2 0RE UK; 7 Present Address: Genentech, South San Francisco, CA USA

**Keywords:** Single-cell CRISPR screen, SNV, GWAS, Base editor, Causal variant, VUS

## Abstract

CRISPR screens with single-cell transcriptomic readouts are a valuable tool to understand the effect of genetic perturbations including single nucleotide variants (SNVs) associated with diseases. Interpretation of these data is currently limited as genotypes cannot be accurately inferred from guide RNA identity alone. scSNV-seq overcomes this limitation by coupling single-cell genotyping and transcriptomics of the same cells enabling accurate and high-throughput screening of SNVs. Analysis of variants across the JAK1 gene with scSNV-seq demonstrates the importance of determining the precise genetic perturbation and accurately classifies clinically observed missense variants into three functional categories: benign, loss of function, and separation of function.

**Supplementary Information:**

The online version contains supplementary material available at 10.1186/s13059-024-03169-y.

## Background

Human genetics, population-scale biobanks, and cancer genome sequencing have identified thousands of genetic variants associated with disease [1, 2]. However, the rate of discovery of such variants vastly exceeds our ability to understand and experimentally model their functional effects.

High-throughput CRISPR-mediated pooled screening for phenotype [3] or coupled to single-cell transcriptomics [4] offers a powerful way to assess the effects of thousands of genetic perturbations. However, it is mainly limited to knockouts or manipulation of expression level using CRISPR interference or CRISPR activation since the guide RNA (gRNA) is used as a proxy of cell genotype and thus the efficiency of the perturbation must be very high. This makes it very challenging to screen for single nucleotide variants, since base editing, prime editing, or homology-directed repair (HDR) efficiency is rarely high enough [5], is highly variable between different genomic sites and cell types, and can lead to undesirable editing byproducts such as bystander mutations, insertions/deletions, or heterozygous edits. Even in those cases where base or prime editor screens have been successful [6–9], it is not possible to distinguish cells containing a non-functional gRNA that has not edited the genome from cells with a functional gRNA that have successfully introduced a benign edit that does not have an effect on cell phenotype. This means that benign variants cannot be accurately classified without simultaneous genotyping of the cells.

It is possible to directly sequence genomic edits during flow cytometric [9] or life-death [10]-based phenotypic selection, allowing SNVs to be screened with these readouts, but this is difficult to apply to transcriptomic readouts. A number of methods have been developed to allow the coupling of the genotype and phenotype of single cells. These fall into two broad categories: those that amplify the whole genome and transcriptome from a single cell [11–17] or those that directly read out genotype from the RNA [18–21]. The first class is often plate-based, limiting their scalability, with the exception of two recent studies that either use split pool barcoding [17] or droplet microfluidics [16] to increase the number of cells that can be assayed. While these techniques are useful for discovering natural variation and its effect on the transcriptome, they are not ideal for perturbation screens due to the cost of whole-genome sequencing and the relatively high allele dropout rate, making it difficult to accurately call SNVs, especially heterozygotes. Even in the best example, allele dropout rates are around 20–25% [13], with high coefficients of variation across the genome, and the higher throughput methods show even higher variability [16]. One method, TARGET-seq [22], uses targeted amplification of DNA and achieves low allele dropout (around 10%), but this is only possible in plates due to the need for a large dilution step after cell lysis and thus not scalable to tens or hundreds of thousands of cells. The second class of methods relies on the direct detection of variants within the RNA, using short [18–20] or long read sequencing [21] to capture variants at different locations within the transcript. While these methods require only limited adaptation of existing protocols and can be high-throughput, they are only possible for genes with high expression levels in order to capture sufficient transcripts from each cell. They are also blind to mutations that lose RNA expression such as nonsense or frameshift mutations that trigger nonsense-mediated decay, and it is difficult to accurately identify heterozygous mutants that show allele-specific expression. Importantly, non-coding variants that are not transcribed, such as those frequently identified from genome-wide association studies, are not accessible to this kind of technology.

To address these limitations in scale, accuracy, and applicability to all SNVs, we developed a method, scSNV-seq, that uses transcribed genetic barcodes to couple targeted single-cell genotyping with transcriptomics to identify the edited genotype and transcriptome of each individual cell rather than predicting genotype from gRNA identity. This enables accurate high-throughput pooled screening for SNVs with single-cell “omics” readouts.

## Results and discussion

We used a previously described [23] cytosine base editor screen in HT-29 cells with gRNAs tiling across the *JAK1* gene to establish our method. We have phenotypic data on the response of each variant to interferon gamma (IFN-γ), which triggers cell death and induction of PD-L1 and MHC-I expression, both of which are blocked by loss of JAK1 function [23]. Interrogated *JAK1* variants can inform the genetic basis of immunological disorders and mechanisms of cancer resistance to anti-tumor immunity.

**Fig. 1:**
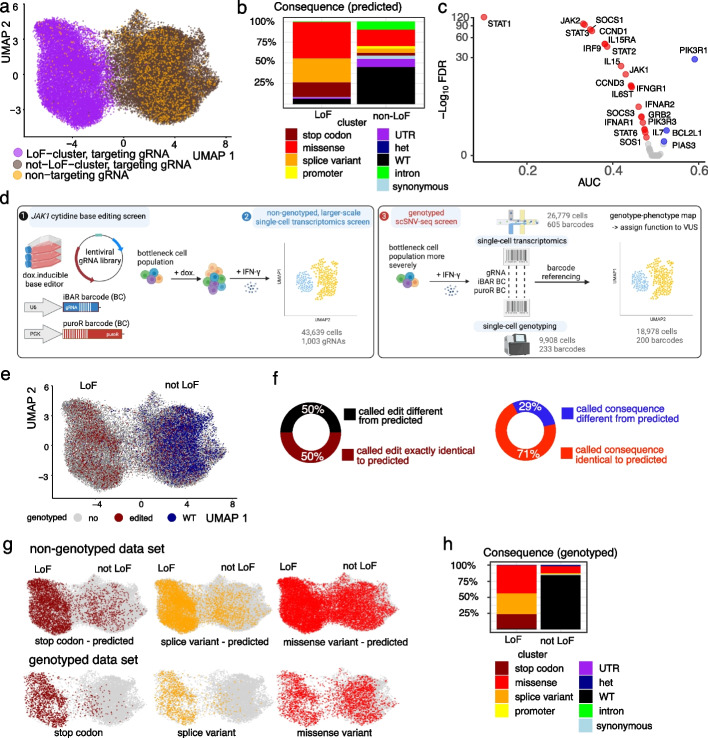
A single-cell base editor screen tiling across *JAK1* is improved by coupling genotype with transcriptome. **a** UMAP of LoF and not LoF meta-clusters for the non-genotyped experiment including all cells with a uniquely assigned gRNA. NT-gRNAs are highlighted in orange. **b** Distribution of consequences of the predicted mutations for each cluster. **c** Differential gene expression analysis of JAK-STAT pathway genes between the LoF cluster and non-targeting gRNAs. AUC < 0.5 indicates downregulation (red, if significant) and AUC > 0.5 upregulation (blue, if significant). **d** Overview of high-throughput SNV phenotyping. Base editing of *JAK1* was achieved through the introduction of a barcoded gRNA library into a doxycycline-inducible cytidine base editor expressing HT-29 cancer cells (left panel, 1). After editing, cells were induced with IFN-γ before single-cell transcriptomics (left panel, 2) or bottlenecked and processed for targeted single-cell DNA sequencing (right panel, 3). Transcriptomes and edited genotypes of single cells were linked through genetic barcodes to assign function to variants of unknown significance (VUS). **e** UMAP combining the non-genotyped (gray) data set with all genotyped cells with confidently called genotype (GT, 18,978 cells). Red and blue indicate edited and wild-type (WT) cells respectively. **f** Percentage of barcodes for which the called homozygous DNA editing is exactly the same as predicted based on complete editing in the window (maroon/black) or for which the functional consequences of the edit on the protein sequence are the same (red/blue). **g** UMAPs highlighting mutational consequences for the predicted genotypes (upper, non-genotyped data set) compared to the called genotypes (lower, genotyped data set). The colored cells are homozygous stop codon (brown), splice (yellow), or missense variants (red), with other cells shown in gray. Compare to Additional file 1: Fig. S2i. **h** Percentages of cells showing the consequence of mutations from actual genotyping in LoF and not LoF clusters. For the assignment of probable consequences using VEP, only homozygous mutations were included, as heterozygous edits are not expected to have a strong functional consequence. See also Additional file 1: Figs. S1 and S2

Single-cell transcriptomics of base edited cells after IFN-γ treatment showed that cells fell into two broad clusters (Fig. 1a). To assign functions to each cluster, we assigned gRNAs to each cell (Additional file 1: Fig. S1a) and predicted the resulting edits (Additional file 1: Fig. S1d). We identified the two clusters as JAK1 loss of function (LoF) or not LoF by merging smaller clusters based on gene expression using the prevalence of cells with non-targeting gRNAs (NT-gRNA) in each cluster (Additional file 1: Fig. S1b, c). Stop codons and splice variants were predominantly contained in the LoF cluster, with WT, synonymous, and intronic variants in the not LoF cluster (Fig. 1b, Additional file 1: Fig. S1e). This classification was confirmed by comparison with the results of previous screens for growth (proliferation score, Additional file 1: Fig. S1f) or induction of PD-L1 and MHC-I (FACS score) in the presence of IFN-γ (Additional file 1: Fig. S1g) [23].

Analysis of differential gene expression between the two clusters showed a strong enrichment for components of the IFN-γ signaling pathway (Fig. 1c), including *JAK1* itself, *IFNGR1*, *JAK2*, *IRF9*, *STAT1*, *STAT2*, and *STAT3*, and downstream effectors such as *IL15*, *IL15R1*, *CCND1*, *CCND3*, and *SOCS3*. *STAT1* was one of the most downregulated transcripts in JAK1 LoF cells, suggesting a positive feedback loop may maintain *STAT1* mRNA expression in the presence of JAK1 signaling [24]. Also, the regulatory subunit of phosphoinositide-3-kinase (PIK3R1) was highly upregulated in the JAK1 LoF cells, consistent with extensive cross-talk between IFN-γ and PI3K signaling pathways [25].

We next performed targeted single-cell genotyping to identify the precise mutations introduced in *JAK1* within each cell. To couple the genotype to the transcriptome, the cells used for this screen had transcribed genetic barcodes introduced by lentivirus on the same vector as the gRNA library (Fig. 1d). We introduced two independent barcodes to compare their effectiveness and to increase the sensitivity of barcode detection. This showed that the majority of cells had both barcodes detectable (Additional file 1: Fig. S2a). One barcode was in the 5′ untranslated region of the puromycin resistance gene (puroR BC), and the second was within the first loop of the gRNA (iBAR BC) [26]. Barcodes were highly complex (the “ Methods” section), and each transduced cell was thus marked with a unique barcode. Both barcodes can be read out in targeted single-cell genotyping simultaneously with amplicons tiling across the JAK1 gene, as well as single-cell transcriptomics using targeted enrichment of the transcribed barcode sequences (the “ Methods” section).

Although our single-cell genotyping method has low allele dropout rates of around 10% [27], there is inherent noise in single-cell genotyping resulting from amplification from only 2 copies of the genome. In order to understand how to accurately genotype these triploid cells, we bottlenecked the population severely to obtain multiple daughter cells from each edited cell, all of which are marked by the same barcode. When analyzing genotyping from single cells, we frequently see multiple heterozygous edits per cell which are not present when looking at the consensus genotype of barcode groups of 3 or more cells (Additional file 1: Fig. S2b). Thus, we believe these are due to errors in the single-cell genotyping, which can be overcome by considering multiple cells within a single barcode group and that we can confidently call genotypes with a minimum of 3 cells per barcode. Based on our data, we would suggest genotyping each barcode across 10 cells to ensure most barcodes have > 3 cells and measuring transcriptome with ~ 50 cells depending on the strength of phenotype meaning that in the order of a thousand variants can be assayed in a single experiment. These variants can be within a single gene or spread across hundreds of sites across the genome.

Using the above criteria, in our data, we were able to call 233 barcodes with confident genotypes that were represented by 18,978 cells in the transcriptomics analysis (average 81 cells/barcode) (the “ Methods” section, Fig. 1d, e), and these barcodes were used in all subsequent analyses. For 25 gRNAs, we saw different barcodes for the same gRNA, resulting from multiple independent editing events (Additional file 1: Fig. S2c). When the actual genotypes were compared with those predicted from the gRNA sequence, only 50% of genotypes were exactly as predicted (Fig. 1f), although this was improved to 71% when analyzed at the protein level due to degeneracy in codon usage (Fig. 1f, Additional file 1: Fig. S2d, 2e). Of the 29% with functional consequences different from the predicted ones, 48.4% had heterozygous edits, 45.2% were unedited, and 6.5% had a different functional consequence. The most frequent edits were homozygous (160 of 233 barcodes) followed by heterozygous edits on 1 (73 barcodes) or 2 alleles (30 barcodes) (Additional file 1: Fig. S2e, 2f). Most homozygous edits were within the predicted base editing window (66%, Additional file 1: Fig. S2g, h), with 8% of these also showing homozygous edits outside the window (Additional file 1: Fig. S2h). These results are important for interpreting base editing screens where genotype is inferred from sgRNA identity, since a large proportion of edits are not as predicted.

Analysis of the transcriptome of these genotyped cells showed that there was an improvement in the classification of stop codon or splice variant mutations into the correct (LoF) cluster and WT cells into the not LoF cluster when considering actual genotypes (Fig. 1g, h), compared to using the gRNA as a proxy of genotype. A small number of cells (56) with stop codon mutations were still assigned to the not LoF cluster. However, when considering barcode groups consisting of > 3 cells, all stop codon mutations are in the LoF cluster (Additional file 1: Fig. S2i). This highlights the benefits of analyzing the data in terms of barcode groups and suggests the incorrectly classified single cells are likely due to misassignment of barcodes in the 10 × experiment. Notably, missense mutations present for a barcode group in the not LoF cluster can be unambiguously defined as mutations that do not result in a loss of JAK1 function, rather than gRNAs that do not edit, and can therefore be used to assign these variants of unknown significance (VUS) as true benign mutations.

**Fig. 2:**
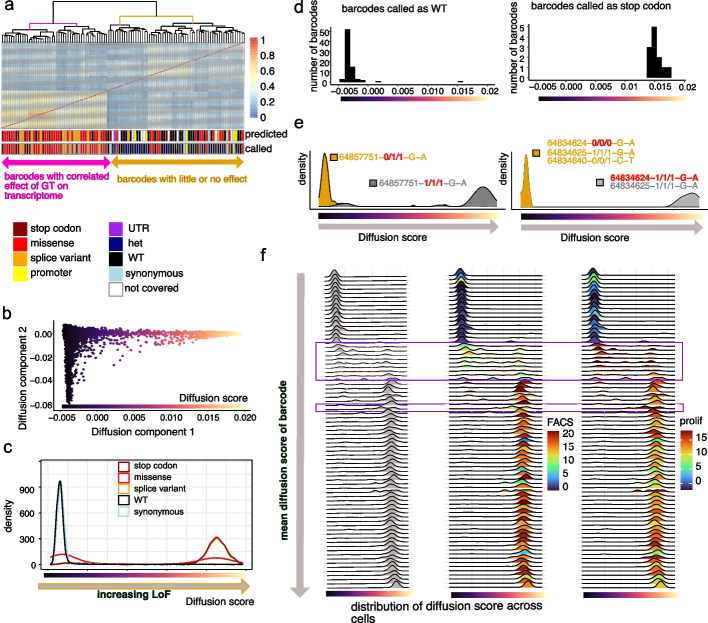
Transcriptomic changes of genotyped cells accurately classify missense mutations into three functional categories. **a** Correlations of differential gene expression of each barcode to cells with WT genotypes, including non-targeting gRNAs. For each barcode, the consequence and predicted consequence of homozygous mutations are shown. The barcodes fall into two groups: one consisting mainly of stop codons, splice variants, and missense mutations and a second one containing many WT barcodes. **b** Diffusion map showing a low-dimensional representation to identify the main directions of variation. **c** The first diffusion component (diffusion score) acts as a measure of loss of function with a high diffusion score for homozygous stop codons and a low diffusion score for WT and homozygous synonymous mutations. **d** Demonstration of low false-negative and false-positive rates for calling edits in barcode groups. Diffusion score for barcodes called homozygous stop codons and WT. **e** Possible phenotypic consequences of small differences in editing. Barcodes with the same gRNA but different edits (heterozygous versus homozygous, one edit versus two consecutive edits). The position is the editing position on chr1. Note that editing may be different for the three alleles of the same cells. **f** Transcriptomic heterogeneity of homozygous missense variants. Density plots for the diffusion scores of all barcodes with homozygous missense variants, including variants with low impact (low diffusion score indicating no LoF-benign), intermediate diffusion scores (indicating separation of function (SoF)), and high impact (high-score missense) mutations. Boxed barcodes highlight variants with intermediate diffusion scores, characterized by lower FACS scores and higher proliferation scores (SoF)

Similarities between the transcriptomic changes resulting from the different mutations separated barcodes into two main groups (Fig. 2a), those containing predominantly LoF mutations (stop codon, splice variant, some missense) or not LoF (WT, synonymous, some missense). We used diffusion maps [28] to identify trajectories in the data (Fig. 2b), and the first diffusion component accurately reflected the trajectory between not LoF and LoF mutations (diffusion score, the “ Methods” section). We confirmed this by comparison with JAK-STAT pathway activity (Additional file 1: Fig. S3a) [29]. The transcriptomic changes caused by the mutations split into two main clusters when ordered by diffusion score (Additional file 1: Fig. S3b) and correlated well with the differential expression of JAK-STAT pathway genes (Additional file 1: Fig. S3c). WT and synonymous variants had very low diffusion scores, stop codon or splice variants had high diffusion scores, and missense mutations were bimodally distributed between the two (Fig. 2c).

Barcodes with genotyped homozygous stop codon mutations were universally (100%, 12 out of 12) classified with high diffusion scores, and all 77 barcodes with WT genotypes except one (> 98%) were classified with low diffusion scores (Fig. 2d). Out of the 15 barcodes called as homozygous splice variants, 93% (14) had high diffusion scores. Therefore, out of the 104 barcodes that were called with either a WT or a definite LoF genotype (stop/splice), 26 were true positives (definite LoF phenotype-high diffusion score), 1 was false positive (called as splice variant, but low diffusion score), one was false negative (precision 96%, recall 96%). This shows that our genotyping pipeline using > 3 cells per barcode is highly effective and has a very low rate of incorrect genotype calls. This compares to 28 predicted stop/splice with 8 false positives and 4 false negatives (precision 78%, recall 88%) using the predicted genotypes.

The benefit of genotyping is illustrated in two examples where we had the same gRNA associated with two different barcodes and where the genotype of these barcodes was different (Fig. 2e). In the first, both barcodes had a homozygous edit at chromosome 1 position 64834625, but only the barcode that was additionally edited at position 64834624 showed a LoF phenotype, indicating that this mutation or the combination of the two together was causing the loss of JAK1 function. In the second example, only the homozygous edit at position 64857751 showed a LoF phenotype, whereas the heterozygous edit did not. Taken together, these observations demonstrate the utility of genotyping editing events to unambiguously interpret variant functions, even in a screen optimized for very high base editing activity.

Some of the missense mutations had a diffusion score between the WT and LoF values, suggesting an intermediate phenotype (Fig. 2c, f). In our previous screen, these gRNAs had strong effects in the proliferation assay (prolif.) but weaker effects on PD-L1 and MHC-I protein expression (FACS, Fig. 2f), suggesting they could be a separation of function (SoF) variants [23]. Closer analysis revealed that cells with these cell barcodes (and thus deriving from the same parent cell) were distributed across the diffusion score range. This shows that for these variants, there is a stochastic response to IFN-γ, with some cells responding as normal, others not at all, and some with an intermediate effect. This may help to explain the difference between their long-term effects on cell growth (prolif, Fig. 2f) and their immediate effects on protein expression (FACS, Fig. 2f), since growth integrates across time, whereas protein expression is a snapshot of their immediate response. SoF variants showed differential expression of IRF9, a key regulator of IFN-γ signaling, that may control the threshold of transcriptional response between WT, SoF, and LoF (Additional file 1: Fig. S3d). These observations would not be possible without genotyping and single-cell analysis.

## Conclusion

In summary, we present scSNV-seq, a technique that allows the direct linkage of genotype to whole-transcriptome readout in high-throughput single-cell perturbation screens. We demonstrate its effectiveness in a base editor mutagenesis screen across *JAK1* to classify LoF missense variants. Importantly, it allows us to identify benign variants or variants with an intermediate phenotype (Additional file 2: Table S1) which would otherwise not be possible. The methodology is applicable to any other methods for introducing variation such as HDR, prime editing [30], or saturation genome editing [10] since it does not rely on gRNA identity to infer genotype. Our method relies on lentiviral barcoding of dividing cells and so cannot be applied to tissue samples or post-mitotic cell types. However, due to the single-cell readout, it can be applied in a cell-type and state-specific manner and to primary cells such as T cells, B cells, hematopoietic stem cells, keratinocytes, and fibroblasts that can be transduced and expanded, but where the inability to clone cells normally prevents analysis of engineered SNVs. The rich phenotypic readout of the whole transcriptome for each perturbation classifies variants based on transcriptional signatures, enabling comparison to perturbations in disease. We believe scSNV-seq will be invaluable for screening the functional significance and downstream effects of the growing list of coding and non-coding variants identified from human genetics analyses such as GWAS and cancer genome sequencing.

## Methods

### gRNA library cloning to include PuroR barcode and iBAR barcode libraries

To introduce the PuroR barcode (in the 5′ UTR of the puromycin resistance gene), a single-stranded ultramer containing NeoUTR3 [31] was amplified using KAPA to add Gibson arms and a 12N barcode in the reverse primer. After SPRI purification, the product was cloned using Gibson assembly into lentivector (Addgene #67,974) cut with XbaI and XhoI. After ethanol precipitation, 5 Gibson reactions were electroporated into supercompetent cells (Endura, Lucigen) and grown in liquid culture to give a coverage of around 100 million barcodes. gRNA with iBAR barcodes were introduced into the PuroR library by amplifying the gRNA library tiling *JAK1* [23] (Twist, 2000 guides, 1055 of which map to JAK1 with the remainder being guides targeting intergenic regions, essential genes, or non-targeting controls) to include a 6N randomized iBAR barcode in the primer. After a nested PCR, the gRNA iBAR library was cloned by Gibson into the PuroR library cut with BbsI and BamHI. After ethanol precipitation, 2 Gibson reactions were transformed into supercompetent cells and grown to give a coverage of around 40 million events. All primers are detailed in Additional file 3: Table S2.

### Base editing screens

For base editing experiments, we derived a clonal line of HT-29 cells expressing a base editor (cytidine BE3-NGG) under a doxycycline-inducible promoter [23] and introduced the lentiviral gRNA library tiling *JAK1* with PuroR and iBAR barcodes as described above. We used an infection rate of ~ 30% to minimize the introduction of multiple gRNAs in one cell and selected infected cells with 2 µg/ml puromycin (Thermo Fisher Scientific). Cells were maintained in 0.5 µg/ml puromycin for the duration of the experiment to maintain gRNA expression. Base editing was induced by the addition of doxycycline (1 μg/ml; Sigma Aldrich) for 72 h. After editing, we bottlenecked a subset of these edited cells (15,000 cells) and also used FACS [23] to select LoF (50,000 cells) to ensure we captured representative phenotypes in our bottlenecked populations. After expansion, these cells were both loaded onto the Chromium X (4 lanes, aiming to recover 60,000 cells per lane) for transcriptomic experiments (see below for further details) and were also further bottlenecked (8000 cells) for the genotyping plus transcriptomic experiments. After further expansion, these cells were single-cell genotyped with the Tapestri machine (Mission Bio, according to the manufacturer’s instructions), using 4 reactions, up to 10,000 cells per reaction and using a custom panel of amplicon sequences (Additional file 3: Table S2) spanning *JAK1* exons and promoter region, as well as the gRNA plus iBAR barcodes and PuroR barcodes. The same population of cells was also loaded onto the Chromium X (2 lanes, aiming to recover 60,000 cells per lane). For all transcriptomics experiments, the base editor was induced again for 24 h as we have found it necessary to have expression of Cas9 to stabilize the gRNA transcripts and improve gRNA detection in single cells. We stimulated cells with IFN-γ (400 U/ml; Thermo Fisher Scientific) for 16 h before processing cells. We used the 5′HT kit (10X Genomics), and cDNA libraries were prepared according to the manufacturer’s instructions. We performed direct gRNA capture by spiking in a scaffold-specific RT primer before loading, and after the cDNA amplification, we performed a nested PCR from the small SPRI fraction to produce a library for sequencing both the gRNA and the iBAR barcode. We also spiked in a puromycin resistance gene-specific RT primer and carried out an analogous nested PCR in order to produce a PuroR barcode library (primer sequences in Additional file 3: Table S2). Sequencing was performed on the NovaSeq 6000 (Illumina).

### Data analysis of single-cell base editor screen without genotyping (non-genotyped large BE experiment)

#### Processing and quality control

We used Cell Ranger 7.0.1 to obtain UMI counts for gRNA and mRNA and for cell calling. For quality control, we removed low outliers for the total count, low outliers for the number of detected features, and high outliers for the percentage of counts from mitochondrial genes using the scater [32] Bioconductor package, obtaining 155,429 cells (non-genotyped large BE experiment).

#### gRNA calling

We developed a robust method to call gRNAs and other barcodes in cells from (UMI) counts using a probabilistic model of mixtures of skewed normal distributions with 3 components. We considered all UMI counts above a minimum threshold of 2 in all cells. Then, we used the mixture model to group them into 3 clusters, 1 cluster for ambient background noise and 2 clusters for signal counts, to allow for a bimodal distribution of signal counts. For robust gRNA assignment and to exclude undetected multiple gRNA assignments in a cell, we defined 2 thresholds for UMI counts: a lower threshold—UMI counts below this threshold mean a 90% probability of being in the ambient cluster—and an upper threshold—UMI counts below this threshold correspond to a 10% probability of being in the ambient cluster. A gRNA was then called in a cell if UMI counts for 1 gRNA are above the upper threshold and no other gRNAs have UMI counts above the lower threshold. We obtained 43,639 cells from this robust assignment of one gRNA and one iBAR per cell, which we used for downstream analysis. Using only cell barcodes with a unique gRNA and iBAR assigned to them also removed most doublets, as these would have 2 gRNAs.

#### Dimensionality reduction and clustering

First, genes that are differentially expressed (DE) for at least one gRNA (with at least ten cells assigned to it) compared to cells with non-targeting gRNAs are identified using the Wilcoxon rank-sum test [33]. Then, we performed principal component analysis (PCA) on the data, subset to the DE genes and the genes in the JAK-STAT pathway. Louvain clustering [34] was performed on a neighborhood graph using the ten nearest neighbors for each cell, based on the low-dimensional representation obtained by the PCA (Additional file 1: Fig. S1b). Two larger meta-clusters (Fig. 1a, referred to as WT (wild-type) and LoF (loss-of-function) are formed by grouping clusters by the similarity of their transcriptomes (see dendrogram in Additional file 1: Fig. S1b) and by the percentage of cells with non-targeting gRNAs in the cluster (Additional file 1: Fig. S1c).

#### Differential expression analysis for LoF gRNAs

gRNAs for which at least 70% and at least 3 cells are in the LoF cluster were assigned to the LoF group. Differential analysis was performed between all cells of the LoF group and all cells with non-targeting gRNAs using the Wilcoxon rank-sum test [33] (Fig. 1c). The Wilcoxon rank-sum test is a standard non-parametric test that compares for each gene how often its expression is higher for the LoF group compared to the cells with non-targeting gRNAs. Genes more highly or lowly expressed significantly often at FDR level of 0.1 are highlighted in Fig. 1c. The area under the curve (AUC) is the proportion of times that the expression of a gene is higher for the LoF group than in a corresponding cell of the non-targeting group, where corresponding refers to being the same quantile within the respective group. Therefore, AUC < 0.5 means downregulation in the LoF group and AUC > 0.5 upregulation. Using a non-parametric approach like AUC is more appropriate and robust for cases where a set of cells cannot be assumed to follow a parametric distribution like a Gaussian or a negative Binomial distribution. Here, we cannot assume cells of the same barcode have been perturbed to follow the parametric distribution, as the cells may have been impacted to different degrees. An extreme example of this is the SoF mutants (Fig. 2f).

### Experiment with genotyping: analysis of scDNA-seq modality

The Tapestri DNA Pipeline On-prem was used for QC, cell barcode correction, alignment, and cell calling, using as the reference the hg38 genome with pKLV2 added (Additional file 3: Table S2). For each cell MissionBio barcode identified as a cell by the pipeline (34,801), variant calling was performed using GATK HaplotypeCaller [35]. gRNA, iBAR, and puroR counts were computed for each cell barcode, using the reads for pKLV2 from the aligned bam files. Then, gRNAs, iBARs, and puroRs were assigned to cells using the same gRNA calling method as described above for the scRNA-seq modality. We obtained 13,102 cells with a unique puroR barcode robustly assigned, 10,869 cells with a gRNA + iBAR combination robustly assigned, and 10,112 cells with both unique puroR and unique gRNA + iBAR assigned, i.e., 77% of cells with a unique puroR barcode assigned were also assigned both gRNA and iBAR, and 93% of all cells with unique gRNA + iBAR were assigned a unique puroR (Additional file 1: Fig. S2a). This showed that while the detection of the puroR barcode was better for the scDNA modality, gRNA + iBAR and puroR assignments agreed almost perfectly for cells with a robust gRNA assignment. It allows us to map puroR barcodes to gRNA + iBAR, to facilitate analysis for the scRNA-seq modality, where we used cells with only iBAR + gRNA assigned and without puroR, as iBAR + gRNA detection was much better than for puroR. We established this correspondence between puroR on the one hand and gRNA + iBAR on the other hand for all puroRs that only occurred paired with one gRNA + iBAR and paired with that gRNA + iBAR for at least 2 cells. By using only cells with confidently assigned unique barcodes, we avoid including doublets and cells with multiple gRNAs, as well as droplets mistakenly identified as cells. Groups of cells from the same parent cell (barcode groups) were identified as groups that either share the same gRNA-iBAR combination and the same puroR. For cases where either of the barcodes could not be called in a cell, the assignment to groups was performed on the basis of the barcode called (iBAR + gRNA or puroR). We obtained 332 unique barcodes with at least 3 cells and with puroR and iBAR + gRNA confidently assigned. The smaller number of gRNAs represented compared to the large BE experiment resulted from deliberate bottlenecking. In fact, only 501 of the gRNAs were present with at least 1 cell for the scDNA modality (290 with at least 2 cells, 184 with at least 10 cells).

Genotypes were then called on a per barcode group basis, to allow robust genotyping for single-cell data, which have higher noise levels than pooled data and may be affected by allele dropout as well as distortion of genotype calling because of ambient counts. First, we subsetted cell genotypes to C- > T and A- > G mutations (for gRNAs on the reverse strand) and removed frequent mutations occurring in more than 10% of the barcodes, as we assumed that they were not caused by the gRNAs.

We called genotypes for barcode groups with at least 3 cells. We used the following computational method to assess for each barcode group whether a genotype can be called robustly (callability) and to call the genotype: For each position in the genome, a variant was called if it was present on at least one allele in at least 2 cells from the group comprising at least 50% of the cells and if a majority of cells with the variant have this variant on the same number of alleles. This relatively low threshold of 50% reflects the fact that it is unlikely that more than 2 cells and more than 50% of the cells of a barcode group have a miscalled mutation by chance and limits the impact of dropout and missed mutations on genotype calling at the level of barcode-groups. A barcode group was called WT, if for each position, no more than 1 cell (or 0 cells if < 10 cells per barcode group) has a mutation on any number of alleles. The accuracy of this approach of genotype calling at the barcode-group level is shown in Fig. 2d. At this level of robustness and accuracy, we were able to call genotypes for 233 barcodes (Fig. 1d, e, Additional file 2: Table S1), out of 332 barcodes with at least 3 cells identified overall (72%), with a total of 9908 cells. For barcodes with at least 3 cells, we found no significant dependence of the callability of the genotype on cell number (Wilcoxon rank sum test, *p* = 10.3%).

Consequences were assigned to edits on the barcode group level using VEP [36], restricting to MANE select proteins. Edits in the *JAK1* promoter region (chr1:64,964,978–64,967,543) were labeled as promoter [23]. For several edits for a genotype, we call the most severe consequence, where stop codon/start lost > splice variant > missense variant > promoter/intron > synonymous. Detailed genotype calls per barcode with consequences and additional analysis results can be found in Additional file 2: Table S1.

### Experiment with genotyping: analysis of scRNA-seq modality

This section describes the process of the scRNA-seq modality for the smaller and bottlenecked experiment that was combined with the genotyping.

#### Basic processing and gRNA calling

Basic processing and gRNA calling were performed in the same way as for the non-genotyped data. iBAR and puroR calling was performed as follows: first, a list of all possible iBARs was created, and a list of puroRs was obtained from the puroR calling at the scDNA level. These lists were used as input in the cellranger pipeline, to obtain UMI counts for iBARs and puroRs in the same way as for gRNAs. Finally, iBARs were called in cells using the same method as for gRNAs. Dimensionality reduction was also performed in the same way as for the non-genotyped data set. We obtained 26,779 cells with a confidently assigned unique gRNA and iBAR. A total of 18,978 of these cells had a iBAR-gRNA combination present among the barcode groups with confident genotype assignment from the DNA modality (200 barcodes, median number of cells per barcode group 14, mean number 95, Fig. 1d).

### Mapping genotypes to the scRNA-seq modality

#### Integration with non-genotyped data set

To compare the genotyped to the larger non-genotyped data set at the level of UMAPs and clusters, we used mutual nearest neighbours [37] for data integration and, based on the integrated PCA representation, assigned to each cell in the genotyped data set the UMAP coordinates of its nearest neighbor in the non-genotyped data set (Fig. 1e), and the most frequent cluster among its 10 nearest neighbors in the non-genotyped data set (Fig. 1h). For the clusters in Fig. 1h, a cell was filtered out if it was the only cell with a specific barcode within a cluster, to denoise possible errors in barcode assignment for the scRNA-seq data.

#### Correlation of differential expression across barcodes

Differential expression was performed for the barcode groups with confidently assigned genotypes and with at least 10 cells for the scRNA-seq modality (114 barcodes). Figure 2a shows the correlations of differential gene expression of each barcode to cells with both WT-genotypes and non-targeting gRNAs. The differential expression compared to the non-targeting cells with WT genotypes was computed for each gene and each barcode with at least 10 cells. Then, we computed the correlation across the AUCs obtained by this differential expression analysis, including the computation of the correlation genes significantly differentially expressed for at least one barcode.

#### Diffusion and pathway scores

Diffusion maps [28] were used to identify trajectories in the data. The first diffusion component, which we identified as the trajectory towards full LoF of JAK1, was named diffusion score. The pathway score for the JAK-STAT pathway (Additional file 1: Fig. S3a) was computed using the PROGENy tool [29].

#### Estimation of false-negative and false-positive genotype calls

We estimated the accuracy of our computational approach to genotyping at the barcode level using stop codons (which we can assume to lead to LoF) and WT (which cannot be LoF). We estimated the number of false positive genotype calls by examining the number of barcodes called as stop codons or splice variants, but with a diffusion score indicative of not LoF. Similarly, false negatives were estimated by considering the number of barcodes called as WT, but with a LoF phenotype (Fig. 2d). False positives and negatives for predicted rather than actually called phenotypes were estimated using predicted genotypes, excluding those gRNAs targeting the JAK1 promoter or UTR region and not covered by an amplicon.

#### Characterization of SoF variants

We explored heterogeneity of LoF level of homozygous missense variants by means of density plots for the diffusion scores of all barcodes with missense variants, including variants with low impact (low diffusion score indicating no LoF benign), intermediate diffusion scores (indicating SoF), and high impact (high-score missense) mutations (Fig. 2f). The plots (one density plot for each barcode) are ordered vertically by the mean diffusion score across the cells with the barcode. Barcodes with intermediate diffusion scores are highlighted by a purple box. A second, smaller, purple box highlights one additional barcode, to illustrate that this barcode has the same genotype as one of the barcodes in the first box. The variants highlighted by the boxes are characterized by lower FACS scores and higher proliferation scores (SoF).

Specific gene regulation differences between SoF and full-impact missense mutations were identified as those either upregulated significantly for SoF compared to full-impact and not downregulated for SoF compared to benign missense variants (AUC > 0.45) or downregulated significantly for SoF compared to full impact and not upregulated for SoF compared to benign missense variants (AUC < 0.55, Additional file 1: Fig. S3d). These cutoffs distinguish these genes from those that are upregulated compared to high-score missense mutations and downregulated compared to benign missense mutations, i.e., their gene expression is on a progressive trajectory between benign and full LoF (area highlighted in yellow in Additional file 1: Fig. S3d).

### Supplementary Information

**Additional file 1.** Supplementary figures and text. Figs. S1, S2 and S3 and legends for Tables S1, S2 and S3.

**Additional file 2: Table S1.** Information associated with individual cellular barcodes.

**Additional file 3: Table S2.** Primer sequences.

**Additional file 4: Table S3.** Sample descriptions and accessions.

**Additional file 5.** Review history.

## Data Availability

The sequencing data sets supporting the conclusions of this article are available in the European Nucleotide Archive (ENA) [38] repository with the accession ERP133355. Sample information and accession numbers are described in Additional file 4: Table S3. Code is available on GitHub [39] (https://github.com/MarioniLab/scSNV-seq) under an open-source GPL-3.0 license and processed data files and the version of the source code used for the manuscript on Zenodo [40] (10.5281/zenodo.10418435) under a CC-BY-4.0 license.
