# Supplementary material for: scSNV-seq: high-throughput phenotyping of single nucleotide variants by coupled single-cell genotyping and transcriptomics
Source: Genome Biol. 2024 Jan 15;25:20. doi: 10.1186/s13059-024-03169-y (PMC10789043; doi:10.1186/s13059-024-03169-y)
Supplement: Supplementary file 1 — Additional file 1. Supplementary figures and text. Figs. S1, S2 and S3 and legends for Tables S1, S2 and S3. [file 13059_2024_3169_MOESM1_ESM.pdf]

Supplementary information for scSNV-seq: high-throughput phenotyping of single nucleotide variants by coupled single-cell genotyping and transcriptomics

Sarah E. Cooper*, Matthew A. Coelho*, Magdalena E. Strauss*, Aleksander M. Gontarczyk, Qianxin Wu, Mathew J. Garnett, John C. Marioni, Andrew R. Bassett

Fig S1

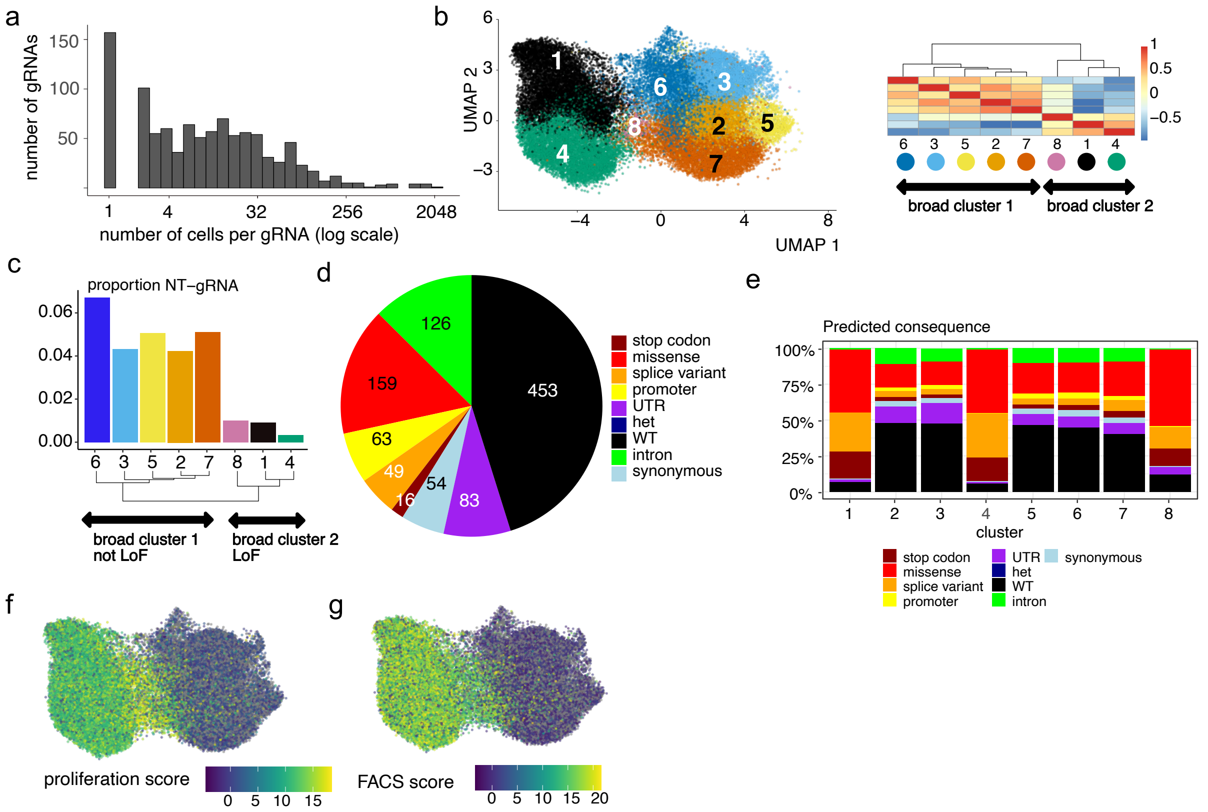

Fig S1. A large single-cell base editor screen tiling across *JAK1* without genotyping.

a) Distribution of numbers of cells per gRNA for the experiment without genotyping. Overall, there are 1,003 gRNAs represented with at least one cell, and the mean number of cells per gRNA is 44.

b) Clusters found by clustering the 43,639 cells with a unique gRNA assigned confidently using Louvain clustering on a neighbourhood graph using 10 nearest neighbours for each cell, represented on the UMAP (left). A dendrogram based on correlation of per-cluster mean gene expression (principal components) shows that there are two groups of clusters (1-4-8; 2-3-5-6-7).

c) Proportion of cells with non-targeting gRNAs groups confirms the two meta-clusters (1-4-8; 2-3-5-6-7).

d) Number of gRNA for each predicted consequence for the non-genotyped experiment, out of a total of 1,003 gRNAs assigned confidently to at least one cell (see also Fig 1d).

e) Distribution of predicted consequence across clusters confirms a structure with two meta-clusters. A cell is assigned the predicted consequence based on complete editing within the editing window of the gRNA.

f) UMAP highlighting proliferation score[22].

g) UMAP highlighting FACS-score[22].

Fig S2

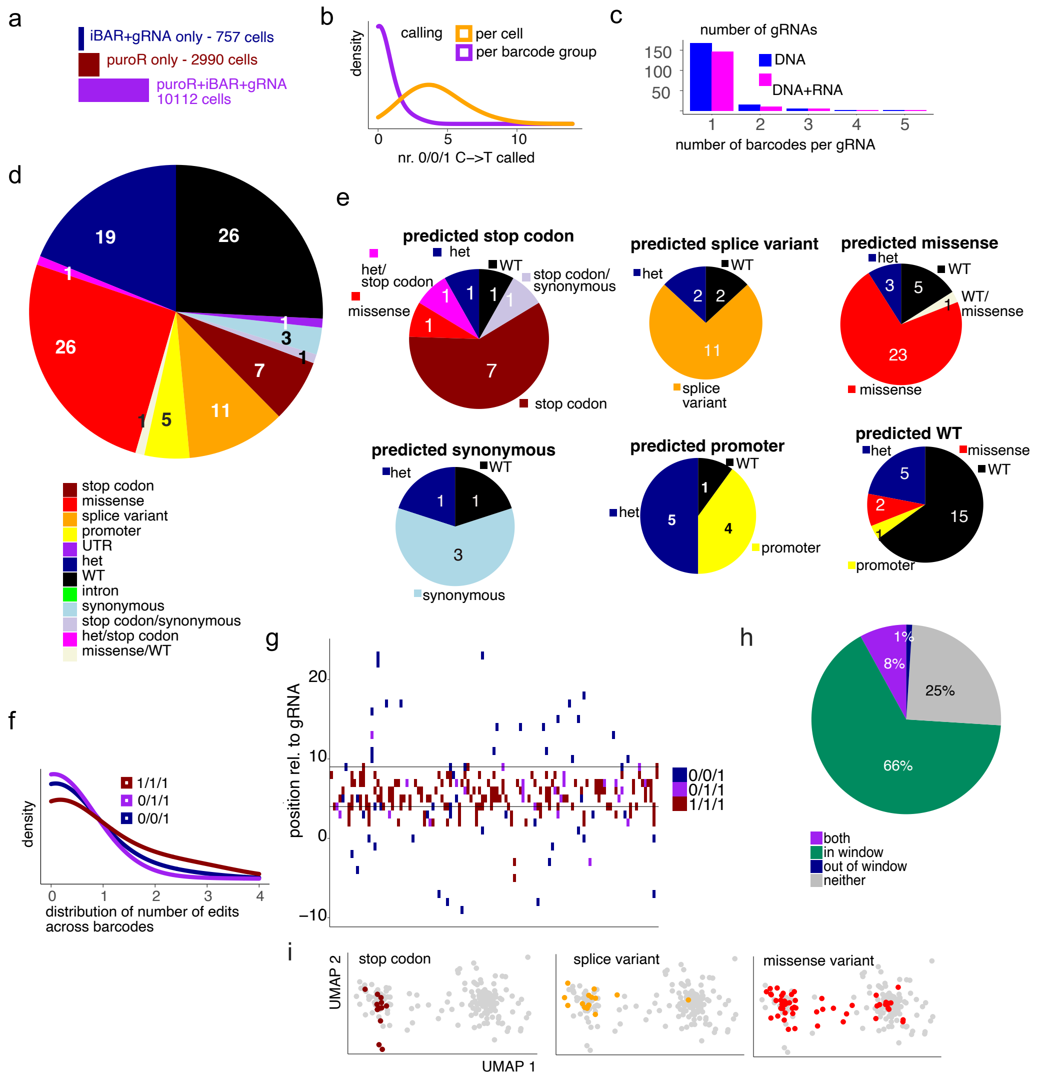

Fig S2. A single-cell base editor screen tiling across *JAK1* is improved by coupling genotype with transcriptome.

a) Number of cells with confident calling of different barcodes for scDNA-seq modality.

b) Multiple cells are required to accurately call genotypes. Edits on one allele (0/0/1) are often called spuriously, when genotype calling is based on a single cell because of noise in single-cell genotyping, with the yellow line showing a mode of four such mutations per cell. After performing barcode group based genotype calling with barcodes with at least 3 cells, such edits were rare (purple line).

c) Number of genotyped puroR/iBAR barcodes per gRNA for the genotyped experiment. Barcodes also present in the RNA modality are shown in magenta, those only present for the DNA in blue.

d) Numbers of gRNAs for the genotyped experiment split by consequence of their homozygous edits. Several consequences split by "/" indicates that there are several different barcodes for the gRNA with different genotypes and different consequence.

e) As d, but performed separately for each predicted consequence. The outcomes labelled as het are from barcode groups without a homozygous edit, which could therefore not be classified in terms of consequence.

f) Distribution of number of edits per cell across the 233 confidently genotyped barcodes, split by zygosity.

g) Position of edits relative to the start of the gRNA for the genotyped barcodes, coloured by zygosity. The expected editing window is marked by horizontal lines.

h) Percentage of homozygous barcode edits within and outside of the 4-9 base editing window, where position 21-23 is the protospacer adjacent motif (PAM), for barcodes with JAK1 targeting gRNAs.

i) UMAP coloured as in Fig 1g for genotyped data set, where each dot represents a barcode group rather than a single cell.

Fig S3

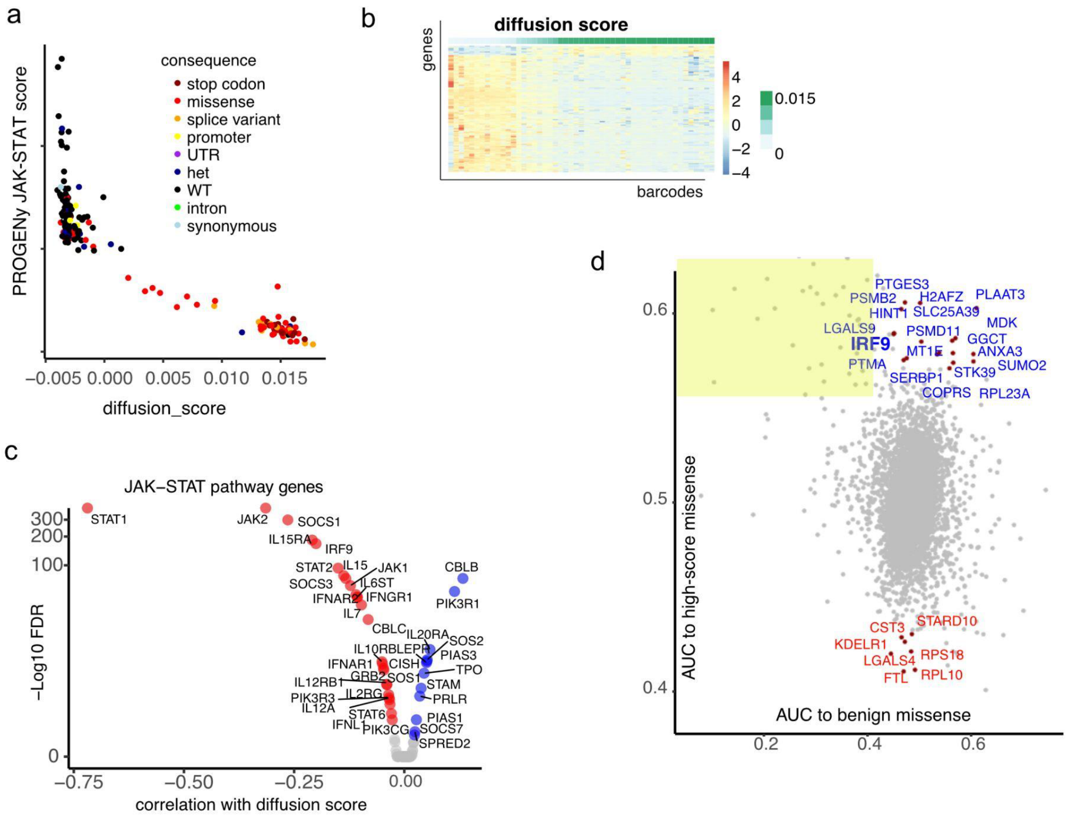

Fig S3. Transcriptomic changes of genotyped cells accurately classify three categories of missense mutations into three functional categories.

a) JAK-STAT pathway activity, measured by the PROGENy score, is negatively correlated with the diffusion score. Each dot is a barcode, coloured by consequence. PROGENy scores are computed per cell, and then averaged across barcodes.

b) Average expression of genes correlated with diffusion pseudotime for barcodes with missense variants, the barcodes ranked by diffusion score.

c) Correlation of JAK-STAT pathway genes with the diffusion score for all barcodes. Significant positive (blue) and negative (red) correlations are highlighted.

d) Genes regulation differences between SoF and full-impact mutations. The plot shows genes that are upregulated for SoF variants compared to high-score missense and not downregulated compared to benign mutations (high AUC to high-score missense, blue), or downregulated for SoF variants compared to high-score missense and not upregulated compared to benign variants (red).

Table S1 - Information associated with individual cellular barcodes

Information on the 233 most confidently called cellular barcodes including identity of puroR and iBAR barcodes (puroBC, gRNA_iBAR), called genotype (GT), presence and nature of homozygous edits (any homozygous edits, homozygous edits), targeted gene, position of gRNA in hg38 (start, strand, end, exonic). It also contains details of the actual consequences (consequence of each individual mutation, worst consequence) from variant effect predictor as well as those predicted from gRNA sequence (predicted consequence). Variants present in the COSMIC database (existing variation) or other databases (citation) are also noted. We show the functional effect of the variant using FACS score and proliferation score[22], the diffusion score (this study), PROGENy pathway score. Information on the guide RNA sequence (gRNA_seq), whether the edit was within the expected editing window (editing positions and out_of_window_edit) and predicted and actual edited sequences (predicted_seq_edited or actual_edit_hom) and whether these were the same (actual_equal_predicted_editing) are also shown.

Table S2 - Primer sequences

The primer sequences (5'-3') used for construction of the gRNA library (library construction), detection of the barcodes in scRNAseq (single-cell barcode detection), for genotyping of the JAK1 gene (JAK1 amplicon panel) and the partial pKLV sequence are indicated.

Table S3 - Sample descriptions and accessions

The sample accessions (SAMPLE TITLE) in the European Nucleotide Archive for study PRJEB48915 and the respective Sanger identifiers (SANGER TUBE ID), short sample name (SAMPLE NAME) and description (SAMPLE DESCRIPTION).
