# Supplementary material for: scSNV-seq: high-throughput phenotyping of single nucleotide variants by coupled single-cell genotyping and transcriptomics
Source: Genome Biol. 2024 Jan 15;25:20. doi: 10.1186/s13059-024-03169-y (PMC10789043; doi:10.1186/s13059-024-03169-y)
Supplement: Supplementary file 5 — Additional file 5. Review history. [file 13059_2024_3169_MOESM5_ESM.docx]

Review History

**First round of review**

**Reviewer 1**

**Were you able to assess all statistics in the manuscript, including the appropriateness of statistical tests used?**

Yes, and I have assessed the statistics in my report.

**Comments to author:**

Cooper, Coelho and Strauss present a method for unambiguously assigning genotype to phenotype at single cell resolution using combined analysis of the transcriptome as a phenotype and using single cell genotyping. This should be a useful approach for understanding gene variants. The drawbacks of the manuscript are that only 1 gene is evaluated, the logic of their use of dual barcodes + clones is a bit confusing and only a limited number of genotypes are analyzed. Overall I think the manuscript needs to make clearer for the reader what the limiting aspects/costs are of this approach as it seems many more cells are sampled than are used in the final analysis. That said I think this manuscript will be of interest to a broad audience and congratulate the authors on a very nice approach.

Main conceptual concerns:
- Please clarify the logic for the average reader of why certain experimental design choices are important/required for unambiguously assigning genotype to phenotype in this approach. E.g. why 2 barcodes and why clones are required for this analysis.
- Please add another figure panel to Figure 1 showing a schematic of the number of cells sampled by phenotype/genotype, the number of genotypes accurately measured from clones, the number of sgRNAs/unique edits that are informative etc. It seems like the authors had to sample a large number of cells to end up with only confident genotype information for 233 barcodes.
- Also please comment on how confidently your phenotypes are measured for genotypes/barcodes that have few or many cells measured. For example you say 233 barcodes with 3 or more cells were further analyzed.
- Please display and discuss how common differential allelic edits (e.g. heterozygous edited alleles) are and how these were filtered out or used at various steps.
- Please clarify how many of the 2000 sgRNAs in the base editing library are analyzed in the final filtered data and discuss or model how this might be scaled up to analyze more genes or more completely analyze all sgRNAs in a library targeting 1 gene.

Figure 1 specific comments:
1A. Labels should be added to 1A, please plot the negative controls cells on the UMAP plot in 1A or S1B as another color distributed across the UMAP
1D. As stated above- it would help the average reader to provide more explanation of the logic of why both barcodes are necessary and/or what information each allows you to assess. Is the combinatorial complexity of using 2 barcodes required to unambiguously say a genotype is unique? Also then why you need to use clones? Is this simply so you can measure the phenotype of multiple cells per genotype without sampling too many cells? Also would be helpful to state that the single cell genotyping uses targeted amplicon sequencing of JAK1 + both barcodes. Please also add more text on the iBAR barcode design, pool complexity and how many unique iBARs are associated with each sgRNA. I realize all of this is to some degree in the methods but I feel it would be helpful for this kind of methods paper to include more detail and logic in the results.

-Also it would be helpful to describe in detail how the analysis deals with allelic heterogeneity - this is discussed later in figure 2 but should be addressed in Figure 1 in my opinion.


1F. Why do you get some number of stop codons in the non LoF cluster?

Figure 2 specific comments:
2A. The way this data is displayed is confusing or not that informative. It is not super clear or quantitative that there are two clusters. Can you illustrate the clusters by hierarchical clustering and adding a dendrogram or some other means of measuring/displaying this?

**Reviewer 2**

**Were you able to assess all statistics in the manuscript, including the appropriateness of statistical tests used?**

Yes, and I have assessed the statistics in my report.

**Comments to author:**

Sarah E. Cooper et al. developed a method called scSNVseq with the help of stably integrated puroR barcode and iBAR barcode in gRNA to couple the genotype to the transcriptome of the same, edited cell at single-cell level. Authors demonstrated that they identified benign variants, loss-of-function variants and variants with an intermediate phenotype in the single-cell base editor screen tiling across *JAK1*, and the classification of scSNVseq data correlated well with previous FACS-based screen or proliferation-based screen.

1, The structure of the manuscript is unconventional, as it has no Introduction and Discussion.

2, The writing in its current format is hard to follow, with an insufficient level of explanation on what has been done for which purpose, what has been observed, and what conclusion has been drawn. For example, it is interesting that the author used two barcodes in each vector. However, there is a lack of explanation on the benefit of this design. Only saying "To unambiguously couple the genotype to the transcriptome" is not very helpful to elaborate on the advantage of this design over the others (e.g., using only gRNA as a barcode (Atray Dixit, 2016, Cell), iBARs (iBARs, Shiyou Zhu, 2019, Genome Biology)). The author should explain the advantages of using two barcodes instead of one. A direct comparison of genotype assignment to scRNA-seq to other methods is also required to demonstrate the advantages.

3, Relevant to the previous point, it is unacceptable that the original iBAR paper was not even cited. Many previous work in this field (connecting genotype with single-cell transcriptome) wasn't properly cited.

4, SIDR (Genome research, 2018) and DEFND-seq (bioRxiv preprint, 2023) have enabled the efficient separation of genomic DNA and total RNA from the same single cell and allowed the direct sequencing of genotype and transcriptome. Identifying SNV from scRNA-seq data has also been reported (Olivier Poirion, 2018, Nature Communications).

5, This method required multiple daughter cells to perform scRNA-seq and single-cell genotyping, respectively, which hinders the application of this technique in clinical settings where the input of cells is quite low.

6, The author bottlenecked a subset of the edited cells before the targeted single-cell DNA sequencing; It is not clear how to ensure this subpopulation of cells can cover the whole population of edited cells for scRNA-seq.

7, The bottleneck process significantly reduced the number of identified barcodes. There are 2000 gRNAs designed to generate variations in the JAK1 gene, but only 233 barcodes(around 200 gRNAs) were called for genotype, which seems insufficient to cover all the designed SNVs across the gene.

1, Fig. 1f, what does the color of the dots indicate? Please clarify the detail of the UMAP plot.

2, Fig. 1a, what are the x-axis and y-axis?

3, In the paragraph "High-throughput CRISPR-mediated pooled screening for phenotype", line 17 is incorrect in grammar.

4, The overall rigid of this manuscript requires significant improvement. Here are just two examples.

(1) In the paragraph "High-throughput CRISPR-mediated pooled screening for phenotype", line 22, The author should clearly define what is "non-function gRNA" and what is "introduced an edit without an effect".

(2) In the paragraph "Single-cell transcriptomics of base edited cells", line 44, it is unclear what does the "high-level identities" mean.

**Authors Response**

**Point-by-point responses to the reviewers’ comments:**

Reviewer #1: Cooper, Coelho and Strauss present a method for unambiguously assigning genotype to phenotype at single cell resolution using combined analysis of the transcriptome as a phenotype and using single cell genotyping. This should be a useful approach for understanding gene variants. The drawbacks of the manuscript are that only 1 gene is evaluated, the logic of their use of dual barcodes + clones is a bit confusing and only a limited number of genotypes are analyzed. Overall I think the manuscript needs to make clearer for the reader what the limiting aspects/costs are of this approach as it seems many more cells are sampled than are used in the final analysis. That said I think this manuscript will be of interest to a broad audience and congratulate the authors on a very nice approach.

We thank the reviewer for their constructive comments, which we believe we have addressed in the substantially revised manuscript.

Main conceptual concerns:
- Please clarify the logic for the average reader of why certain experimental design choices are important/required for unambiguously assigning genotype to phenotype in this approach. E.g. why 2 barcodes and why clones are required for this analysis.

We apologise that this was not clear, and have clarified the logic of using two barcodes in the text and new Supp Fig. 2a, namely to assess how well each worked and to increase detectability of the barcodes. We have also replaced the clone terminology, since this is misleading and replaced with barcode. The barcodes are marking the daughter cells of a parental editing event and are thus marking cell clones within the population. We have also clarified that there is inherent noise in single cell genotyping due to amplification from only two copies of the genome, and thus we need multiple daughter cells of a parental event in order to accurately call genotypes.

- Please add another figure panel to Figure 1 showing a schematic of the number of cells sampled by phenotype/genotype, the number of genotypes accurately measured from clones, the number of sgRNAs/unique edits that are informative etc. It seems like the authors had to sample a large number of cells to end up with only confident genotype information for 233 barcodes.

We have now added in the cell and barcode numbers at each step in the process to a new figure 1d and clarified this in the text. The low number of barcodes is partly by design through bottlenecking the cell population, since we wanted to over-sample (i.e. to obtain more cells per barcode) to establish the scale of experiments. It is also partly due to inefficiencies and losses throughout the process. The revised text clarifies these points.
We have also discussed the scale of experiment that would be possible, which we believe is up to 1000 variants per experiment. The costs are difficult to estimate because of variation in DNA capture efficiency in MissionBio, bottleneck size and thus distribution of barcodes, editing efficiencies, and discounts on consumables, each of which has a large impact on the cost of an experiment. We therefore believe it is not helpful to be too explicit in these numbers.

- Also please comment on how confidently your phenotypes are measured for genotypes/barcodes that have few or many cells measured. For example you say 233 barcodes with 3 or more cells were further analyzed.

We have now discussed this more in the main text and methods, and added in new supplementary figures (new Supp Fig. 2b and 2i) to explain these results. Due to the noise inherent in single cell genotyping due to amplification from only two copies of the DNA, we observed that mutations on a single allele (“heterozygotes”) were very common when analysing the genotype from a single cell (Supp Fig 2b). These were virtually removed when we analysed the consensus genotype from 3 cells for a particular barcode, showing that these are indeed noise and that 3 cells were sufficient to accurately call genotypes.
Similarly, we show that we can remove the incorrectly assigned cells (e.g. stop codon mutations in the non-LoF cluster) observed in figure 1g at the single cell level by taking the average from 3 or more cells (new Supp Fig. 2i). We analysed whether increasing cell numbers beyond 3 cells per barcode improved genotype calling but found no significant improvement (Wilcoxon rank sum test, p=10.3%).
In terms of phenotyping, the transcriptional signature resulting from JAK1 knockout is very strong, and so we need very few cells per barcode in the single cell transcriptomics in order to accurately call their functional effect. However, the number of cells required in other situations will depend on the effect size, and thus could vary from tens to hundreds depending on the strength of the phenotype.

- Please display and discuss how common differential allelic edits (e.g. heterozygous edited alleles) are and how these were filtered out or used at various steps.

We have discussed this point further in the revised text and Supp Fig. 2d, e, f, g and h. In general, the most frequent edits were homozygous edits (total of 160 across the 233 genotyped barcodes), followed by edits on one allele (a total of 73 across the 233 barcodes), and on two alleles (a total of 30), with many cells having several homozygous edits (Supp Fig. 2f). We have added this information to the text.
Only homozygous edits show an effect on the phenotype, so the functional consequences are only shown for homozygotes. We have modified the legends for Supp Fig. 2d,e,h make this clear. For the comparison of actual and predicted editing (Fig. 1f, Supp Fig. 2h), the predictions assumed that all Cs in the base editing window were edited on all alleles, which we mention in the text.

- Please clarify how many of the 2000 sgRNAs in the base editing library are analyzed in the final filtered data and discuss or model how this might be scaled up to analyze more genes or more completely analyze all sgRNAs in a library targeting 1 gene.

We have clarified the number of barcodes analysed in each experiment in the revised Fig 1d. We have also clarified in the methods that only 1,055 of the 2,000 sgRNAs target the JAK1 gene, with the remainder being controls targeting intergenic regions, essential genes and non-targeting guides.
In this proof-of-principle paper, where we were establishing the parameters, we were not aiming to cover the entire population of edited cells, and as you point out we analysed 233 barcodes corresponding to 192 sgRNAs. We bottlenecked to a greater extent in order to get more cells per barcode in both the genotyping and transcriptomics so that we could understand the number of cells that were required. This has shown that we can have as few as 3 cells per barcode for genotyping and 50 cells per barcode for the transcriptomics, meaning that we can sample up to around 1000 genotypes per experiment. Because the primers for the genotyping can simultaneously target 500 different locations anywhere in the genome, these variants can be spread across a single or multiple genes. We have added this logic into the revised manuscript, and made this clear in the revised figure 1d.

Figure 1 specific comments:
1A. Labels should be added to 1A, please plot the negative controls cells on the UMAP plot in 1A or S1B as another color distributed across the UMAP

We have now corrected this figure and coloured the negative controls in Fig 1A.

1D. As stated above- it would help the average reader to provide more explanation of the logic of why both barcodes are necessary and/or what information each allows you to assess. Is the combinatorial complexity of using 2 barcodes required to unambiguously say a genotype is unique? Also then why you need to use clones? Is this simply so you can measure the phenotype of multiple cells per genotype without sampling too many cells? Also would be helpful to state that the single cell genotyping uses targeted amplicon sequencing of JAK1 + both barcodes. Please also add more text on the iBAR barcode design, pool complexity and how many unique iBARs are associated with each sgRNA. I realize all of this is to some degree in the methods but I feel it would be helpful for this kind of methods paper to include more detail and logic in the results.

As mentioned above, we have now revised the text to clarify these points in both the main text and methods.

-Also it would be helpful to describe in detail how the analysis deals with allelic heterogeneity - this is discussed later in figure 2 but should be addressed in Figure 1 in my opinion.

As mentioned above, we have now extended this discussion and moved it to an earlier point in the text along with moving Figure 2e to Figure 1g, which we agree makes this clearer.

1F. Why do you get some number of stop codons in the non LoF cluster?

We have further analysed this, and discuss this in the revised text and new Supp Fig 2i. Whilst we do see a small number of single cells (56) with stop codon mutations assigned to the not LoF cluster (Fig 1g), when we consider the average position of a barcode, all stop codons are correctly classified (Supp Fig 2i). This highlights the benefits of analysing the data in terms of barcode groups, and suggests the incorrectly classified single cells are likely due misassignment of barcodes in the 10x experiment.

Figure 2 specific comments:
2A. The way this data is displayed is confusing or not that informative. It is not super clear or quantitative that there are two clusters. Can you illustrate the clusters by hierarchical clustering and adding a dendrogram or some other means of measuring/displaying this?

We have added a dendrogram to Fig 2A and clarified in the text.

Reviewer #2: ===

Sarah E. Cooper et al. developed a method called scSNVseq with the help of stably integrated puroR barcode and iBAR barcode in gRNA to couple the genotype to the transcriptome of the same, edited cell at single-cell level. Authors demonstrated that they identified benign variants, loss-of-function variants and variants with an intermediate phenotype in the single-cell base editor screen tiling across *JAK1*, and the classification of scSNVseq data correlated well with previous FACS-based screen or proliferation-based screen.

We thank the reviewer for their constructive comments on our manuscript.

1, The structure of the manuscript is unconventional, as it has no Introduction and Discussion.

We have now re-formatted the manuscript in line with the requirements of a short report at Genome Biology.

2, The writing in its current format is hard to follow, with an insufficient level of explanation on what has been done for which purpose, what has been observed, and what conclusion has been drawn. For example, it is interesting that the author used two barcodes in each vector. However, there is a lack of explanation on the benefit of this design. Only saying "To unambiguously couple the genotype to the transcriptome" is not very helpful to elaborate on the advantage of this design over the others (e.g., using only gRNA as a barcode (Atray Dixit, 2016, Cell), iBARs (iBARs, Shiyou Zhu, 2019, Genome Biology)). The author should explain the advantages of using two barcodes instead of one. A direct comparison of genotype assignment to scRNA-seq to other methods is also required to demonstrate the advantages.

We apologise that this was unclear, and have now clarified this in the substantially revised text. For instance, we have clarified the logic of using two barcodes in the text and new Supp Fig. 2a, namely to assess how well each worked and to increase detectability of the barcodes. We have also added in a new section comparing our methodology to other existing methods in the literature discussing advantages and limitations during the introduction and discussion.

3, Relevant to the previous point, it is unacceptable that the original iBAR paper was not even cited. Many previous work in this field (connecting genotype with single-cell transcriptome) wasn't properly cited.

We apologise for this oversight, and have now cited the iBAR paper as well as a number of other papers describing similar technologies for single cell genotyping and transcriptomics which are also discussed in the revised text.

4, SIDR (Genome research, 2018) and DEFND-seq (bioRxiv preprint, 2023) have enabled the efficient separation of genomic DNA and total RNA from the same single cell and allowed the direct sequencing of genotype and transcriptome. Identifying SNV from scRNA-seq data has also been reported (Olivier Poirion, 2018, Nature Communications).

We have now included a discussion of these papers and a number of others that we think are relevant in a new section in the introduction and discussion of the revised text. This includes how they compare to our own methodology making the advantages and limitations clear.

5, This method required multiple daughter cells to perform scRNA-seq and single-cell genotyping, respectively, which hinders the application of this technique in clinical settings where the input of cells is quite low.

We agree with the reviewer that it is not possible to use this method for cells that cannot be cultured in vitro since both editing and barcoding would be impossible. However, we do believe it is very useful for primary cells that can be cultured, but where clonal lines would be impossible to generate. For instance in T cells, B cells, HSCs, etc. it has not been possible to look at CRISPR edited SNV mutations unless the efficiency of mutations is near 100% which is rarely the case for SNVs. We have discussed these advantages and limitations in the revised discussion.

6, The author bottlenecked a subset of the edited cells before the targeted single-cell DNA sequencing; It is not clear how to ensure this subpopulation of cells can cover the whole population of edited cells for scRNA-seq.

In this proof-of-principle experiment, where we are establishing the parameters, we were not aiming to cover the entire population of edited cells. Indeed, we bottlenecked to a greater extent in order to get more cells per barcode in both the genotyping and transcriptomics so that we could understand the number of cells that were required. This has shown that we can have as few as 3 cells per barcode for genotyping and 50 cells per barcode for the transcriptomics, meaning that we can sample up to 1000 genotypes per experiment in the future. We have added this logic into the revised manuscript, and made this clear in the revised figure 1d.

7, The bottleneck process significantly reduced the number of identified barcodes. There are 2000 gRNAs designed to generate variations in the JAK1 gene, but only 233 barcodes(around 200 gRNAs) were called for genotype, which seems insufficient to cover all the designed SNVs across the gene.

As above, we were not aiming to cover all possible variants with this experiment, but have now included a discussion of scaling. We have now clarified the number of barcodes analysed in each experiment in the revised Fig 1d.
We have also clarified in the methods that only 1,055 of the 2,000 sgRNAs in the library target the JAK1 gene, with the remainder being controls targeting intergenic regions, essential genes and non-targeting guides.

1, Fig. 1f, what does the color of the dots indicate? Please clarify the detail of the UMAP plot.

We have revised the legend accordingly.

2, Fig. 1a, what are the x-axis and y-axis?

We have now labelled this figure.

3, In the paragraph "High-throughput CRISPR-mediated pooled screening for phenotype", line 17 is incorrect in grammar.

We have reworded and clarified this sentence in the revised text.

4, The overall rigid of this manuscript requires significant improvement. Here are just two examples.
(1) In the paragraph "High-throughput CRISPR-mediated pooled screening for phenotype", line 22, The author should clearly define what is "non-function gRNA" and what is "introduced an edit without an effect".

We have altered the wording substantially throughout the manuscript to clarify various points including this one. Non-functional refers to gRNAs that do not edit, whereas an edit without an effect is a benign SNV. Benign SNVs have been successfully introduced and genotyped, but result in WT-like JAK1.

(2) In the paragraph "Single-cell transcriptomics of base edited cells", line 44, it is unclear what does the "high-level identities" mean.

We have re-worded this sentence to clarify this point.

**Second round of review**

**Reviewer 1**

the authors have addressed all of my concerns related to their publication of the scSNV-seq method.

minor comment-
-please keep line weights the same in Figure 2 (e.g. Figure 2A dendrogram and Figure 2C missense)

**Reviewer 2**

The authors have substantially revised the manuscript and improved the logic flow as well as the clarity.
However, as a methodology paper, it is still highly recommended to compare the scSNV-seq with 1-2 existing methods to demonstrate the advantage of this methodology.
